# Case of Phlegmasia Dolens

**Published:** 1819-03

**Authors:** Thomas Ingram Rayner


*For the London Medical and Physical Journal.*

### *Case of Phlegmasia Dolens;* by THOMAS INGRAM RAYNER, Esq.

I WAS called to see Sarah Field, (residing a little distance from Wakefield), who was delivered of her first child three weeks before, and who had got what her attendants termed a swelled leg: they had fomented it with warm water, by advice of the former medical attendant. When I saw her, the left leg and thigh were twice their natural size, of a deadly white colour, but very hot and dry to the touch; and she complained of a great deal of pain in the left labium pudendi, where it began first, and, thence proceeding down the limb, contracting the muscles on the inner side of the thigh. There was great irritation in the system; the appetite was impaired; the pulse quick, and the tongue parched and brown.

I ordered her ten grains of pulvis ipecacuanhæ compositus at bed-time, and to take plentifully of diluting liquors; and, in the morning, a saline purgative of magnes. sulphas. ℥i. liq. menthæ sativæ, ℥viii. three table-spoonfuls to be taken every three hours; and the limb to be rubbed with camphor ℥ſs. dissolved in ol. olivæ, ℥v. night and morning; after which it was to be wrapped in a flannel bandage. These measures answered better than I expected; for in three days the limb, from the foot to the knee, was not above its natural size, and the patient's general health was nearly restored. In a fortnight's time, she had perfectly recovered her usual health, the thigh only being a little larger than common, but which subsided in the course of a week.

*Barston-square, Wakefield;*
*Sept.* 4, 1818.

